# Correction: Survival of infants ≤24 months of age with brain tumors: A population-based study using the SEER database

**DOI:** 10.1371/journal.pone.0224570

**Published:** 2019-10-24

**Authors:** Claire Faltermeier, Timothy Chai, Sharjeel Syed, Nathan Lau, Lior Elkaim, George Ibrahim, Anthony Wang, Alexander Weil, Anne Bendel, Aria Fallah, Albert Tu

The first two authors, Claire Faltermeier and Timothy Chai, should be noted as contributing equally to this work.
