# Supplementary material for: Survival of infants ≤24 months of age with brain tumors: A population-based study using the SEER database
Source: PLoS One. 2019 Sep 25;14(9):e0223051. doi: 10.1371/journal.pone.0223051 (PMC6760899; doi:10.1371/journal.pone.0223051)
Supplement: Attachment — Submitted filename: SEER paper review 2019 infant brain tumors.pdf [file pone.0223051.s009.pdf]

Feltermeier et al reviewed the files of 2996 affected children, aged 24 months and younger, with brain tumors from the Surveillance, Epidemiology, and End Result (SEER) database between 1973 and 2013. The authors concluded that overall survival for infants with brain tumors has improved from the 1970's, with the exception of choroid plexus and embryonal tumors. They present data on "1) distribution of infantile brain tumors, 2) survival strings for brain tumors by subtype and epoch, and 3) trends in treatment and shifts in therapeutic approaches over time." The authors cite their prior work describing the SEER database and add some intrinsic limitations with the SEER database for these specific questions, most importantly the quantification of extent of resection and granular data on outcomes, especially important in pediatrics. Overall this is very carefully thought out work that adds to the literature by stratifying this rare patient population by histological diagnosis, addressing the important improvements in outcomes, correlating with changes of care over time.
